# Inclusion Biogenesis and Reactivation of Persistent *Chlamydia trachomatis* Requires Host Cell Sphingolipid Biosynthesis

**DOI:** 10.1371/journal.ppat.1000664

**Published:** 2009-11-20

**Authors:** D. Kesley Robertson, Ling Gu, Regina K. Rowe, Wandy L. Beatty

**Affiliations:** Department of Molecular Microbiology, Washington University School of Medicine, St. Louis, Missouri, United States of America; Yale University School of Medicine, United States of America

## Abstract

Chlamydiae are obligate intracellular pathogens that must coordinate the acquisition of host cell-derived biosynthetic constituents essential for bacterial survival. Purified chlamydiae contain several lipids that are typically found in eukaryotes, implying the translocation of host cell lipids to the chlamydial vacuole. Acquisition and incorporation of sphingomyelin occurs subsequent to transport from Golgi-derived exocytic vesicles, with possible intermediate transport through endosomal multivesicular bodies. Eukaryotic host cell-derived sphingomyelin is essential for intracellular growth of *Chlamydia trachomatis*, but the precise role of this lipid in development has not been delineated. The present study identifies specific phenotypic effects on inclusion membrane biogenesis and stability consequent to conditions of sphingomyelin deficiency. Culturing infected cells in the presence of inhibitors of serine palmitoyltransferase, the first enzyme in the biosynthetic pathway of host cell sphingomyelin, resulted in loss of inclusion membrane integrity with subsequent disruption in normal chlamydial inclusion development. Surprisingly, this was accompanied by premature redifferentiation to and release of infectious elementary bodies. Homotypic fusion of inclusions was also disrupted under conditions of sphingolipid deficiency. In addition, host cell sphingomyelin synthesis was essential for inclusion membrane stability and expansion that is vital to reactivation of persistent chlamydial infection. The present study implicates both the Golgi apparatus and multivesicular bodies as key sources of host-derived lipids, with multivesicular bodies being essential for normal inclusion development and reactivation of persistent *C. trachomatis* infection.

## Author Summary

The genus *Chlamydia* is composed of a group of obligate intracellular bacterial pathogens that cause several human diseases of medical significance. *C. trachomatis* is the most commonly encountered sexually transmitted pathogen, as well as the leading cause of preventable blindness worldwide. The prevalence of chlamydial infections, and the extraordinary morbidity and health care costs associated with chronic persisting disease, justifies the research efforts in this area of microbial pathogenesis. Despite their clinical importance, the mechanisms by which these intracellular bacteria obtain nutrients essential to their growth remain enigmatic. Acquisition of sphingolipids, from the cells that chlamydiae infect, is essential for bacterial propagation. This study identifies a requirement for the lipid sphingomyelin from the infected host cell for bacterial replication during infection, and for long-term subsistence in persistent chlamydial infection. Blockage of sphingomyelin acquisition results in premature release of bacteria, a reduced bacterial number, and failure of the bacteria to cause a persisting infection. In this study, we have identified and subsequently disrupted specific sphingomyelin transport pathways, providing important implications on therapeutic intervention targeting this successful microbial pathogen.

## Introduction

The genus *Chlamydia* is composed of obligate intracellular prokaryotic pathogens that cause a range of clinical sequelae in humans encompassing ocular, genital, and respiratory tract infections. Consequences of subsequent chronic disease include blindness, infertility, arthritis, and possible coronary heart disease [1],[2]. Despite their notoriety clinically, the molecular interactions between *Chlamydia* and its host cell that allow for propagation, persistence, and subsequent pathology, remain elusive. The defining biological characteristic of these successful pathogens is a unique process of intracellular development, with an infectious elementary body (EB) initiating uptake into a target host cell. The chlamydial EB subsequently differentiates to the noninfectious, metabolically active reticulate body (RB) within the confines of a membrane-bound vacuole termed an inclusion. Successive growth and replication, giving rise to a large inclusion body containing a multitude of infectious EBs, is contingent upon the acquisition of biosynthetic constituents from the nutrient-rich host cell cytosol. In response to nutrient or immunological stress [3], Chlamydiae can also enter into a persistent phase of development characterized by morphologically altered RBs that can be maintained intracellularly for extended periods of time. Alternating infectious and persistent phases of chlamydial growth correlate with acute and chronic infections *in vivo* [4]. The cellular biosynthetic constituents that sustain persistent chlamydiae, and allow for emergence from a persistent state, are poorly understood.

The intricacies of this host-pathogen interaction, which allow for acquisition of biosynthetic precursors from the host cell, remain largely undefined. Vacuole-bound chlamydiae attain nucleotides, amino acids, and lipids from the host cell [5]. Eukaryotic-derived phospholipids, sphingomyelin, and cholesterol are found within purified chlamydiae, suggesting that these host-derived constituents traverse the inclusion membrane with subsequent incorporation into the bacterium [6],[7]. Translocation of lipid droplets to the chlamydial inclusion lumen represents one potential source of neutral lipids [8],[9]. Host cell sphingolipids are required for the intracellular growth of *C. trachomatis* [10], with sphingomyelin attained via the intersection of the chlamydial inclusion with Golgi-derived exocytic vesicles destined for the plasma membrane [11]–[13]. Multivesicular bodies (MVBs), late endocytic organelles abundant in sphingolipids and central to intracellular lipid segregation, also serve as a source for host-derived lipids and a potential intermediate in Golgi to inclusion transport [14],[15]. To further delineate lipid acquisition pathways pirated by the chlamydial inclusion, specific inhibitors of host cell lipid biosynthesis and/or trafficking were evaluated for their effects on chlamydial growth and inclusion development. The present study focuses on sphingomyelin biosynthesis, a host cell pathway validated as essential for growth and replication of chlamydiae by Engel and colleagues [10]. Our studies indicate that sphingomyelin biosynthesis is requisite to inclusion membrane biogenesis and stability, and demonstrate that MVBs are a major source for this essential lipid.

## Results

### Inhibition of host cell sphingomyelin biosynthesis results in loss of inclusion membrane integrity

**Figure 1:**
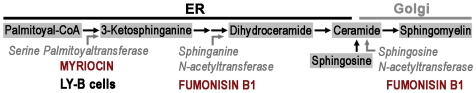
The sphingomyelin biosynthetic pathway. The precursors of sphingomyelin are synthesized in the endoplasmic reticulum with subsequent transfer of ceramide to the Golgi apparatus, the site of the final step in sphingomyelin biosynthesis. The targets for the sphingomyelin inhibitors myriocin and fumonsin B1 are indicated, as well as, the site of enzymatic deficiency of LY-B cells.

**Figure 2:**
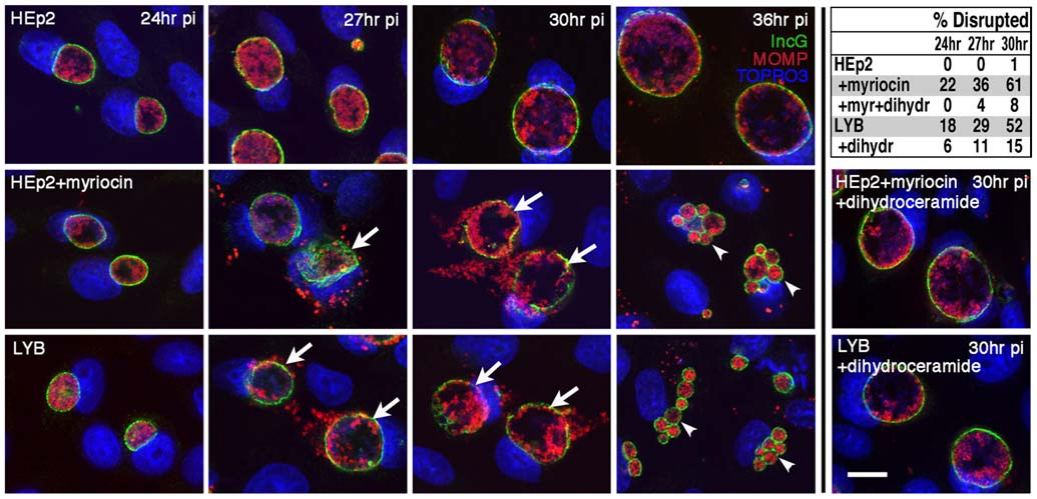
Inhibition in sphingomyelin biosynthesis results in the disruption of inclusion stability. HEp-2 or LY-B cells were infected with *C. trachomatis* E (MOI 0.2) and treated with 25 µM myriocin at 1 hr pi where indicated. Infected cells were fixed at 24, 27, 30, and 36 hr pi and subsequently immunolabeled with anti-incG antibody (anti-rabbit Alexa Fluor 488) and anti-MOMP antibody (anti-mouse Alexa Fluor 568) to precisely identify the boundary of the chlamydial inclusion and the intrainclusion bacteria, respectively. TOPRO-3 labeling was used to identify both intracellular bacteria and the host cell nuclei. Analysis of 0.5 µm confocal optical sections of infected cells revealed disruption of inclusion integrity in HEp-2 cells treated with myriocin, or in SPT-deficient LY-B cells (indicated by white arrows). Disruption of inclusions resulted in early lysis of infected cells and reinfection evident at 36 hr pi (indicated by white arrowheads identifying multiple inclusions). *Lower right panels*: Supplementing the culture medium with 5 µM dihydroceramide for 48 hr prior to infection of HEp-2 or LY-B cells reversed the inhibitory effect of SPT inactivity. Scale bar = 20 µm. *Upper right table*: The percent of disrupted inclusions was quantitated for the cells and treatment conditions indicated.

Specific inhibitors of sphingomyelin biosynthesis and trafficking were evaluated for effects on chlamydial growth and inclusion development. Treatment of infected cells with 25 µM myriocin, a potent inhibitor of serine palmitoyltransferase (SPT), the initial enzyme in the biosynthesis of sphingomyelin (Figure 1) [16], revealed striking morphological alterations in inclusion maturation. Confocal analysis of untreated *Chlamydia*-infected cells revealed normal inclusion development with the vacuole expanding in size from 24 to 36 hr postinfection (pi) (Figure 2). Infected cells cultured in the presence of myriocin, revealed a marked loss of inclusion membrane integrity with disruption of the inclusion and release of intracellular bacteria, initially evident at 24 hr pi (22% of infected cells with disrupted inclusions) and most notable at 30 hr pi (61%) (Figure 2). At 36 hr pi, myriocin-treated cells contained small multiple inclusions of heterogeneous size, rather than the large single inclusion typical of untreated cells (Figure 2). The concentration of myriocin used in these studies had no effect on host cell viability.

The CHO-K1 mutant cell line, LY-B [17], which contains a mutation in the *LCB1* gene and therefore does not express SPT, was used to independently test the role of sphingomyelin. *C. trachomatis* inclusions in LY-B cells showed a collapse of membrane integrity, similar to myriocin treatment (Figure 2). In addition, at 36 hr pi, LY-B-infected cells contained small multiple inclusions comparable to those observed in myriocin-treated HEp-2 cells. The complemented cell line, LY-B/LCB1, supported normal inclusion development comparable to that observed in both CHO-K1 and HEp-2 cells (data not shown), confirming that maintenance of inclusion membrane integrity was dependent on host cell SPT activity.

To confirm that the loss of inclusion membrane integrity was a consequence of a deficiency in host cell sphingomyelin rather than an indirect effect of depleted SPT activity, cells were cultured in the presence of 5 µM dihydroceramide or 5 µM sphingosine prior to infection. Dihydroceramide and sphingosine are precursors of sphingomyelin, positioned downstream of SPT, allowing for the restoration of sphingomyelin synthesis under conditions of SPT inactivity (Figure 1) [10],[18]. These sphingomyelin precursors reversed the detrimental effects of SPT-deficiency in LY-B cells or myriocin-treated HEp-2 cells, with growth and expansion of intact inclusions morphologically comparable to those present in untreated control cells at 24 to 36 hr pi (Figure 2) (data for sphingosine not shown).

### Inhibition of host cell sphingomyelin biosynthesis results in early redifferentiation and premature release of infectious chlamydiae

**Figure 3:**
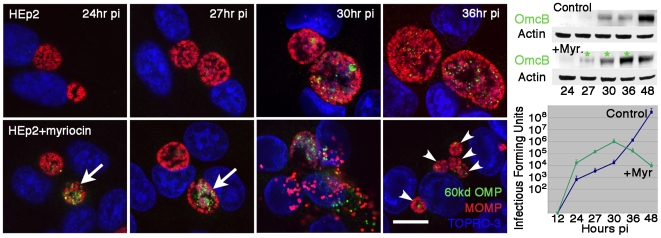
Inhibition in sphingomyelin biosynthesis results in the early redifferentiation of RBs to infectious EBs. HEp-2 were infected with *C. trachomatis* E (MOI 0.2) and treated with 25 µM myriocin at 1 hr pi where indicated. Infected cells were fixed at 24, 27, 30, and 36 hr pi and subsequently immunolabeled with anti-OmcB antibody (anti-rabbit Alexa Fluor 488) and anti-MOMP antibody (anti-mouse Alexa Fluor 568). TOPRO-3 labeling was used to identify both intracellular bacteria and the host cell nuclei. Analysis of 0.5 µm confocal optical sections of infected cells revealed early redifferentiation from RBs to EBs as indicated by the presence of OmcB specific for EBs in myriocin-treated cells (indicated by white arrows). Disruption of inclusions resulted in early lysis of infected cells and reinfection evident at 36 hr pi (indicated by white arrowheads identifying multiple inclusions). Scale bar = 20 µm. *Upper right panel*: Western blot analysis of OmcB levels in untreated control and myriocin-treated cells revealed the early emergence of OmcB in myriocin-treated cells (indicated by asterisks). Analysis of actin levels served as a loading control. *Lower right panel*: Recovery of infectious *Chlamydia* from untreated control and myriocin-treated cells. Data are presented as mean infectious forming units of triplicate cultures+/−s.e.m.

The intracellular developmental cycle of *C. trachomatis* E requires approximately 72 hr to complete, with redifferentiation of RBs to infectious EBs occurring prior to release of infectious progeny. At 24 to 36 hr pi, the expanding inclusion contains predominantly noninfectious RBs that, if released indiscriminately from the infected cell, are incapable of initiating an infectious cycle. The presence of multiple small inclusions at 36 hr pi, under conditions of disrupted host cell sphingomyelin biosynthesis, suggested premature release of infectious progeny and subsequent reinfection. To analyze possible early emergence of infectious EBs, the expression of OmcB, an EB-specific protein detectable late in the developmental cycle, was analyzed. In untreated cells, low levels of OmcB were evident at 30 to 36 hr pi (Figure 3), with peak levels emerging at 48 to 72 hr as inclusions reached maximal size and approached lysis (not shown). Myriocin treatment resulted in expression of OmcB as early as 24 hr pi with EBs being dispersed upon premature loss of both inclusion and host cell membrane integrity (Figure 3). Infected SPT-deficient LY-B cells also displayed early emergence of OmcB-positive EBs, temporally similar to those observed under conditions of myriocin treatment (not shown). Western blot analysis confirmed the higher levels of OmcB at 27–36 hr pi in infected cells treated with myriocin as compared to control cells (Figure 3). In addition, higher levels of infectious progeny were released from myriocin-treated cells versus control cells at early times post infection (Figure 3). Collectively, these results indicate that the absence of sphingomyelin results in loss of inclusion membrane integrity, early redifferentiation, and premature release of infectious chlamydiae.

### Host cell-derived sphingomyelin is required for homotypic fusion of inclusions

**Figure 4:**
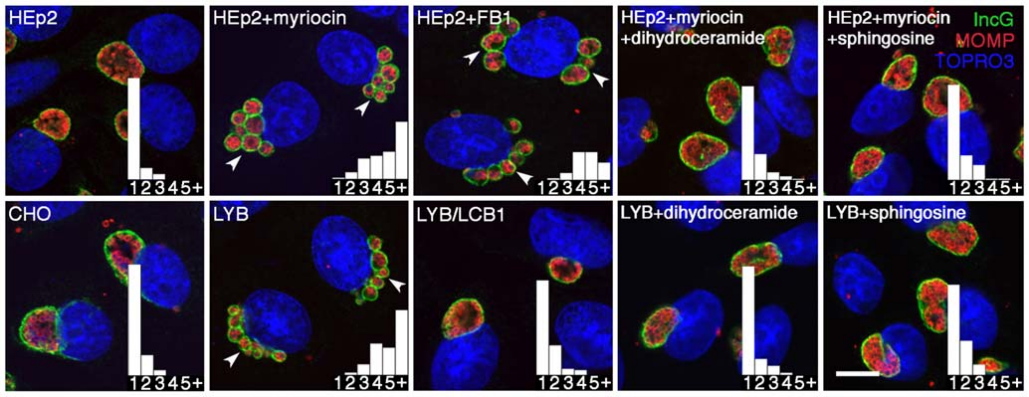
Host cell sphingomyelin-deficiency results in the inhibition of homotypic fusion of inclusions. HEp-2, CHO-K1, LY-B, and LY-B/LCB1 cells were infected with *C. trachomatis* E (MOI 5) and treated with 25 µM myriocin or 5 µg/ml fumonisin B1 at 1 hr pi where indicated. Infected cells were fixed at 16 hr pi and subsequently immunolabeled with anti-incG antibody (anti-rabbit Alexa Fluor 488) and anti-MOMP antibody (anti-mouse Alexa Fluor 568) to precisely identify the boundary of the chlamydial inclusion and the intrainclusion bacteria, respectively. TOPRO-3 labeling was used to identify both intracellular bacteria and the host cell nuclei. Analysis of 0.5 µm confocal optical sections of infected cells revealed the inhibition of fusion of multiple inclusions to a single inclusion in HEp-2 cells treated with myriocin or fumonisin B1, or in SPT-deficient LY-B cells (indicated by white arrowheads). *Right panels*: Supplementing the culture medium with 5 µM dihydroceramide or 5 µM sphingosine for 48 hr prior to infection of HEp-2 or LY-B cells reversed the inhibitor effect of SPT inactivity. The relative number of inclusions per infected cell is shown in graph inserts. Scale bar = 20 µm.

A distinguishing trait of prototypic *C. trachomatis* strains is homotypic fusion of inclusions [19]. Infecting a single cell with multiple EBs of a defined serovar, results in multiple bacterial-containing vacuoles that fuse early in the developmental cycle to form a single inclusion. The presence of multiple inclusions at 36 hr pi in sphingomyelin-depleted cells, suggests reinfection with subsequent disruption of homotypic fusion. To analyze the effect of sphingomyelin deficiency on homotypic fusion, cells were infected with a high MOI of five bacteria per cell and inclusion numbers were determined at 16 hr pi (Figure 4). HEp-2 and CHO-K1 cells generally contained a single inclusion per infected cell as shown in the histogram inserts. HEp-2 cells cultured in the presence of 25 µM myriocin or 5 µg/ml fumonisin B1 (a potent inhibitor of sphingonine and sphinosine N-acetyltransferase, Figure 1), or the SPT-deficient LY-B cells, revealed multiple inclusions per cell. Complementation of the LY-B cells with the *LCB1* gene, resulted in the restoration of host cell sphingomyelin biosynthesis, and the recovery of the inclusion fusion phenotype as shown by a single inclusion per infected cell (Figure 4). To confirm that lack of inclusion fusion was a consequence of a deficiency in host cell sphingomyelin rather than an indirect effect of depleted SPT activity, cells were cultured in the presence of dihydroceramide and sphingosine prior to infection. These sphingomyelin precursors restored the fusion capability to infected cells cultured under conditions of SPT-deficiency with a majority of cells containing a single inclusion (Figure 4). Collectively, these findings indicate that host cell sphingomyelin biosynthesis is required for homotypic fusion of chlamydia inclusions within a single infected cell.

### Host cell-derived sphingomyelin is required for reactivation of persistent chlamydial infection

**Figure 5:**
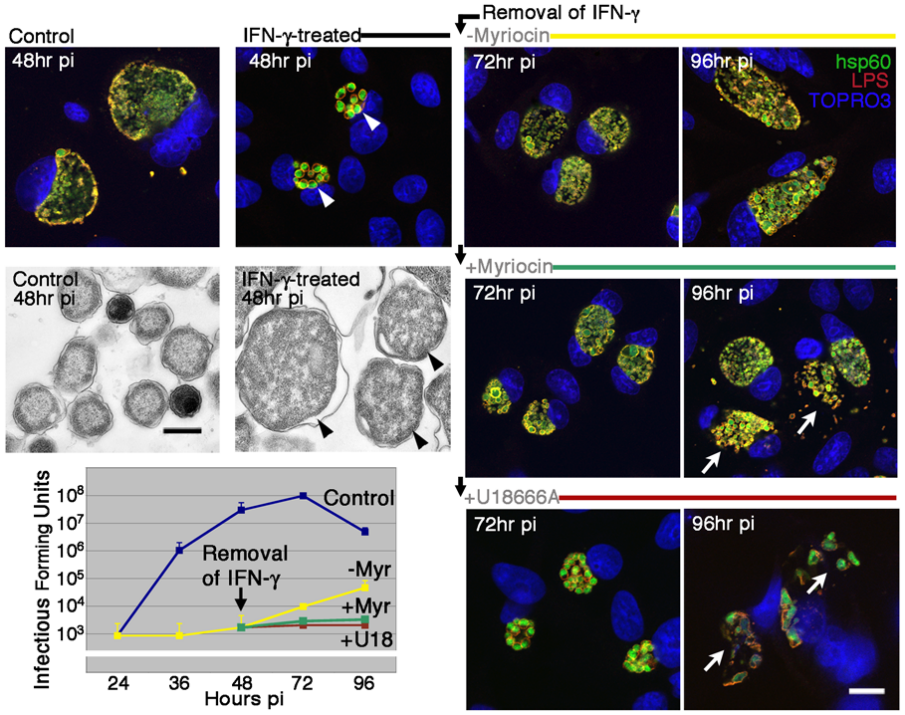
Host cell sphingomyelin-deficiency results in inhibition of reactivation of persistent chlamydial infection. HEp-2 cells were treated with 1 ng/ml IFN-γ where indicated, for 48 hr prior to and 1 hr after infecting with *C. trachomatis* B (MOI 0.2). At 48 hr pi, the IFN-γ was removed and replaced with fresh culture medium with or without myriocin (25 µM) or U18666A (10 µM), and cultured until 96 hr pi. At the indicated times pi, infected cells were immunolabeled with anti-hsp60 antibody (anti-rabbit Alexa Fluor 488) and anti-LPS antibody (anti-mouse Alexa Fluor 568). TOPRO-3 labeling was used to identify both intracellular bacteria and the host cell nuclei. *Upper left panels*: Analysis of 0.5 µm confocal optical sections and electron micrographs of infected cells revealed aberrant, persistent chlamydial development when cultured in the presence of IFN-γ (indicated by arrowheads). *Upper right and center panels*: Analysis by confocal microscopy revealed inclusion expansion with normal inclusion development when cultured in the absence of myriocin but disruption of inclusion integrity when cultured in the presence of myriocin (indicated by white arrows). *Lower right panels*: Culturing IFN-γ-induced persistent chlamydiae in the presence of U18666A revealed a lack of inclusion expansion and loss of inclusion membrane integrity (indicated by white arrows). Scale bar of fluorescent images = 20 µm. Scale bar of electron micrographs = 0.5 µm. *Lower left panel*: The effect of indicated culture conditions on the recovery of infectious organisms. Data are presented as mean infectious forming units of triplicate cultures+/−s.e.m.

Persistence is a hallmark of natural chlamydial diseases, and is characterized by the retention of nonreplicating, aberrant reticulate bodies within the host cell for extended periods of time [3]. Host cell sphingomyelin biosynthesis is essential for maintenance of inclusion integrity during normal chlamydial development, and is likely essential during reactivation of persistent infection, a process concurrent with inclusion membrane expansion. The role of host cell sphingomyelin was tested in a model system of IFN-γ-induced persistence [20]. HEp-2 cells were infected with *C. trachomatis* B, a strain sensitive to IFN-γ-mediated alterations in intracellular growth [21]. Untreated *Chlamydia*-infected cells revealed normal inclusion development with large inclusions at 48 hr pi, while IFN-γ-treated cells harbored smaller inclusions containing enlarged RBs as confirmed by fluorescence and electron microscopy (Figure 5). The persistent state was reversible as shown by the expansion of the inclusion and reactivation of infectious EBs following removal of IFN at 48 hr pi and culturing in fresh medium for an additional 48 hr (Figure 5). In contrast, culturing in the presence of myriocin during the recovery phase resulted in disruption in inclusion membrane integrity and failure of persistent forms to completely reactivate to infectious EBs (Figure 5). These results were confirmed in an alternate *in vitro* model system of penicillin-induced persistence [22],[23]. In *C. trachomatis* serovar B- and servovar E-infected cells treated with penicillin to induce aberrant, persistent chlamydial development, the presence of myriocin during the recovery phase prevented the recovery of infectious EBs (not shown). These studies implicate host cell-derived sphingomyelin as an essential component for maintenance of inclusion membrane integrity during reactivation of persistent chlamydial infection.

### Trafficking of host cell sphingomyelin from the Golgi or MVBs is required for homotypic fusion and normal inclusion development

**Figure 6:**
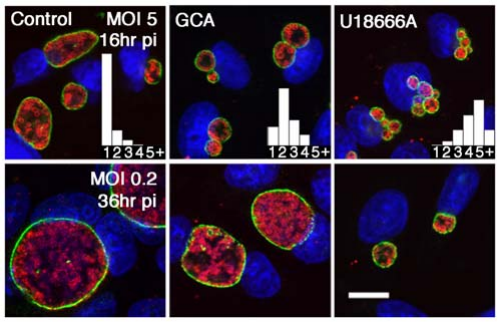
Inhibition of Golgi or MVB transport interrupts homotypic fusion and normal inclusion development. HEp-2 cells were infected with *C. trachomatis* E (MOI 0.2 or 5) and treated with GCA (10 µM) or U18666A (10 µM) at 1 hr pi where indicated. Infected cells were fixed at 16 or 36 hr pi and subsequently immunolabeled with anti-incG antibody (anti-rabbit Alexa Fluor 488) and anti-MOMP antibody (anti-mouse Alexa Fluor 568) to precisely identify the boundary of the chlamydial inclusion and the intrainclusion bacteria, respectively. TOPRO-3 labeling was used to identify both intracellular bacteria and the host cell nuclei. Analysis of 0.5 µm confocal optical sections of infected cells revealed the effects of inhibitors on homotypic inclusion fusion *(upper panels)* or normal inclusion development *(lower panels)*. The relative number of inclusions per infected cell is shown in graph inserts *(upper panels)*. Scale bar = 20 µm.

The precursors of sphingomyelin are synthesized in the endoplasmic reticulum with subsequent transfer of ceramide to the Golgi apparatus, the site of the final step in sphingomyelin biosynthesis (Figure 1). Hackstadt and colleagues have demonstrated the transport of sphingomyelin from the Golgi to the chlamydial inclusion, with incorporation of the sphingolipid into the inclusion membrane and the cell wall of chlamydiae [12],[13]. MVBs, late endocytic organelles abundant in sphingomyelin, have been proposed to provide essential lipids to the chlamydial inclusion and may be an intermediate in Golgi to inclusion transport [14],[15]. To decipher the source of *Chlamydia*-acquired sphingomyelin, the phenotypic effects of inhibitors of Golgi and MVB transport on inclusion maturation were compared to inclusion development under conditions of sphingomyelin deficiency. The inhibitors were used at concentrations that disrupt transport of ceramide-derived sphingomyelin from the Golgi apparatus to the chlamydial inclusion, but have no effect on host cell viability [13],[14]. HEp-2 cells were infected with a high MOI of five bacteria per cell and treated with the indicated inhibitors at 1 hr pi, then analyzed for homotypic fusion at 16 hr pi (Figure 6). Control cells generally contained a single inclusion per infected cell as shown in the histogram inserts. HEp-2 cells were cultured in the presence of golgicide A (GCA), a potent, highly specific inhibitor of GBR1 (Golgi BFA resistence factor 1) that disrupts both anterograde and retrograde transport through the Golgi [24]. GCA-treatment revealed a slight disruption in vacuole fusion with a mean of 2.6 inclusions per infected cell (Figure 6), with a similar result observed upon treatment with 1 µg/ml brefeldin A (BFA) another inhibitor of Golgi function [25] (not shown). HEp-2 cells cultured in the presence of 10 µM U18666A, a pharmacological agent that disrupts trafficking from MVBs [26]–[28], revealed multiple inclusions per infected cell (Figure 6), similar to the conditions of sphingomyelin deficiency (Figure 4). Therefore, interruption of sphingomyelin trafficking from the Golgi delayed inclusion fusion, while a block in MVB trafficking completely impeded fusion, implicating MVBs, an organelle abundant in sphingolipids, as a principle source of chlamydiae-acquired sphingomyelin.

To analyze the effect of inhibitors on inclusion maturation, HEp-2 cells were infected with a low MOI of *C. trachomatis* E, treated with the indicated inhibitors at 1 hr pi and analyzed at 36 hr pi. Confocal analysis of GCA-treated *Chlamydia*-infected cells revealed a slight delay in inclusion maturation with smaller inclusions compared to those in untreated control cells (Figure 6). There was no evidence of inclusion membrane instability as observed under conditions of sphingomyelin deficiency (Figure 2), indicating that sphingolipids may be acquired from an alternate source such as MVBs. Infected cells cultured in the presence of the MVB inhibitor U18666A, revealed a dramatic interruption in inclusion development with significantly smaller inclusions (Figure 6). There was no evidence of inclusion membrane instability as observed under conditions of sphingomyelin deficiency (Figure 2). However, the complete interruption in RB division and subsequent inclusion expansion, implicates additional MVB-derived constituents necessary for normal chlamydial inclusion expansion and development.

### Trafficking of host cell sphingomyelin from MVBs is required for reactivation of persistent chlamydial infection

Host cell sphingomyelin biosynthesis is essential for maintenance of membrane integrity during expansion of the inclusion following reactivation of persistent infection (Figure 5). Because trafficking from MVBs was essential to sphingomyelin-dependent inclusion expansion, the potential significance of these sphingolipid-rich organelles in reactivation of persistent infection was analyzed. Following induction of the persistent state by IFN-γ treatment for 48 hr, removal of IFN and subsequent culturing in the presence of the MVB inhibitor U18666A for an additional 48 hr, resulted in a lack of inclusion expansion, disruption in inclusion membrane integrity, and complete failure of aberrant persistent forms to reactivate to infectious EBs (Figure 5). These studies implicate MVB-derived sphingomyelin, and potentially other MVB constituents, requisite to inclusion membrane integrity during reactivation of persistent chlamydial infection.

## Discussion

The present studies were initiated to identify lipid biosynthetic and transport pathways essential to the intracellular propagation of chlamydiae. These studies revealed novel effects on the intracellular development of chlamydiae under conditions that inhibit sphingomyelin biosynthesis. As demonstrated in classic studies by Hackstadt and colleagues, sphingomyelin synthesized in the Golgi apparatus is transported from the *trans*-Golgi to the chlamydial inclusion with successive incorporation into the bacterial cell wall [12],[13]. In subsequent studies by Engel and colleagues, host cell-derived sphingomyelin was shown to be essential for intracellular development of *C. trachomatis* and optimal production of infectious progeny [10]. In the present study, we further explore this requirement and demonstrate that sphingomyelin biosynthesis is necessary for stability and expansion of the inclusion membrane during both normal intracellular development and reactivation of persistent infection. Blockage of this pathway results in premature egress, reduced bacterial output, and failure to emerge from a persistent state. Hence, disruption of lipid trafficking may provide a novel means to thwart intracellular pathogens.

Chlamydiae undergo their entire intracellular developmental cycle within an inclusion that is bound by a membrane, providing a protected intracellular environment for bacterial replication. Treatment of infected cells with myriocin interrupted inclusion membrane functionality, with complete disruption of membrane integrity resulting in premature dispersal of intracellular bacteria from their protected niche (Figure 2). Myriocin is a potent inhibitor of SPT, the initial enzyme in sphingomyelin biosynthesis (Figure 1) [16]. Analysis of inclusion development in SPT-deficient LY-B cells, and under conditions of concurrent pretreatment with precursors of sphingomyelin, revealed that the compromise in inclusion membrane integrity was a direct result of host cell sphingomyelin deficiency (Figure 2). Actin and intermediate filaments have been shown to stabilize the chlamydial inclusion, with disruption of these host cytoskeletal structures resulting in loss of inclusion membrane integrity and release of bacteria into the host cell cytosol [29]. In the present studies, immunofluorescence analyses of actin and intermediate filaments of both uninfected and chlamydiae-infected cells revealed no obvious morphological alterations in the cytoskeletal structure upon inhibition of sphingomyelin biosynthesis (data not shown).

The disruption of inclusion membrane integrity under conditions of sphingomyelin deficiency occurred concomitantly with the early redifferentiation of noninfectious RBs to infectious EBs (Figure 3). This implies that the procurement of host cell sphingomyelin may be required for inclusion membrane expansion and stability, and programmed conversion to infectious forms. The signals that trigger the replicative RBs to convert to infectious EBs remain elusive. However, it is clear that this developmental transformation coincides with a contact-dependent interaction of the type III secretion (TTS) system with the inclusion membrane. RBs amass at the periphery of the inclusion, with projections of the TTS system mediating intimate contact between the bacteria and the inner face of the inclusion membrane [30],[31]. The proposed chlamydial injectisome acts as a molecular syringe, translocating effector proteins directly from the intrainclusion chlamydiae to the host cell cytosol [32]. This association may be requisite to RB replication and potentially inclusion expansion allowing for nutrient acquisition from the host cell cytosol [33]. The physical detachment of RBs from the inclusion membrane, coupled to inactivation of TTS, signals the initation of late redifferentiation [32]. In the present studies, lipid deprivation may signal the loss of TTS intimate contact and RB detachment leading to premature conversion of RBs to infectious EBs. Host cell-derived sphingomyelin associates transiently with the chlamydial inclusion membrane and incorporates into the bacterial cell wall [12]. Failure of this sphingolipid to incorporate into the inclusion membrane may cause the normally contiguously intact membrane to become indiscriminately permeable to environmental changes that potentially signal RB to EB conversion. Alternately, incorporation of sphingomyelin into the chlamydial cell wall may be essential to RB division and proliferation, with lack of available sphingomyelin being a potential cue for premature redifferentiation.

A secondary function of the inclusion membrane of *C. trachomatis*, distinct from inclusion membrane integrity, is homotypic fusion of multiple inclusions to a single vacuole in multiply-infected cells. The resulting multiple inclusions with greater surface area would require more lipid incorporation into the chlamydial inclusion membrane, indicating that early in infection other host cell lipids are available for incorporation into the expanding inclusion under conditions of sphingomyelin deficiency. Fusion of inclusions is a temperature-dependent process that requires export of the chlamydial incA protein to the inclusion membrane [34],[35]. Characteristic homotypic fusion of inclusions was interrupted when multiply-infected cells were cultured in the presence of myriocin (Figure 4). Analysis of the fusion of multiple inclusions in SPT-deficient LY-B cells, and under conditions of concurrent pretreatment with precursors of sphingomyelin, revealed that the disruption in homotypic fusion was a direct result of host cell sphingomyelin deficiency (Figure 4). These studies did not reveal an alteration in IncA incorporation into the inclusion membrane under conditions of sphingomyelin deficiency, implicating a role for host cell sphingolipids in homotypic fusion independent of incA. Culturing *C. trachomatis*-infected cells under conditions of sphingomyelin deficiency has two distinct phenotypic effects on chlamydial inclusion biogenesis. Interruption in homotypic fusion is observed early in chlamydial inclusion development, while a compromise in inclusion membrane integrity occurs later. These distinct anomalies may result from the failure of sphingomyelin incorporation into the inclusion membrane, implicating a direct role for host cell lipid in maintaining normal inclusion functionality. However, the effect of sphingomyelin deficiency on other lipid biosynthetic or signaling pathways that indirectly alter inclusion biogenesis cannot be disregarded.

Further studies determined the source of sphingomyelin essential to inclusion biogenesis, which includes membrane stability and the capacity for homotypic fusion. As described previously, inhibition of sphingomyelin transport from the Golgi apparatus using the inhibitor BFA, results in smaller, compact inclusions that retain a burst size comparable to untreated controls [12]. In the present studies, this observation was reproduced using both BFA and GCA. In addition, treatment of infected cells with concentrations of BFA or GCA that prevent the incorporation of newly synthesized Golgi-derived sphingomyelin into the chlamydial inclusion, failed to completely disrupt inclusion fusion or inclusion membrane integrity (Figure 6). This implicates another source of sphingomyelin available to the chlamydial inclusion under conditions of disrupted Golgi transport. These studies identify MVBs, late endocytic organelles abundant in sphingolipids and pivotal for intracellular distribution, as a potential source of sphingomyelin essential to homotypic fusion and maintenance of inclusion membrane integrity. U18666A treatment of infected cells, utilizing concentrations that block MVB transport and prevent the incorporation of newly synthesized Golgi-derived sphingomyelin into the chlamydial inclusion [14],[15], revealed complete inhibition of homotypic fusion of inclusions (Figure 6). These findings were identical to the disruption of inclusion fusion observed under conditions of sphingomyelin deficiency (Figure 4). However, inhibition of MVB transport had much more profound effects on RB division and normal inclusion development than what was observed under conditions of sphingomyelin deficiency. A deficit in host cell sphingomyelin resulted in RB division and the expansion of the chlamydial inclusion to a moderate size with subsequent loss of inclusion membrane integrity at 24 to 36 hr pi (Figure 2). In contrast, interruption in MVB transport impeded early RB division and inclusion membrane expansion at a stage in development prior to imposing stress on inclusion membrane integrity. Collectively these studies implicate sphingomyelin, and potentially additional constituents derived from MVBs, essential for inclusion expansion during normal development and the reactivation of persistent *C. trachomatis* infection. However, a pleiotropic effect of inhibitors of MVB transport, on cellular function or potential acquisition of sphingomyelin from alternate sources, cannot be disregarded.

Within the confines of a protected intracellular environment, chlamydiae coordinate the expansion of the inclusion and acquisition of biosynthetic constituents from the host cell cytosol. In the presence of eukaryotic protein synthesis inhibitors, intracellular development proceeds normally, indicating that inclusion expansion may be linked to host cell lipid biosynthesis. These studies identify host cell sphingomyelin biosynthesis as a requisite to *C. trachomatis* inclusion membrane biogenesis and functionality. This encompasses inclusion membrane expansion, homotypic fusion, and stability during normal inclusion development and reactivation of a persistent chlamydial infection. In addition, identification of potential sphingomyelin transport pathways may have important implications when deciphering this unique host-pathogen interaction.

## Materials and Methods

### Antibodies and reagents

Rabbit anti-incG was kindly provided by Dr. Ted Hackstadt (Rocky Mountain Laboratories, NIH, NIAID, Hamilton, MT). Rabbit anti-outer membrane complex protein B (OmcB) was generously provided by Dr. Thomas Hatch (University of Tennessee Health Science Center, Memphis, TN). Monoclonal antibody (mAb) L2I-10 to the major outer membrane protein (MOMP) of *C. trachomatis*, was kindly provided by Dr. Harlan Caldwell (Rocky Mountain Laboratories, NIH, NIAID, Hamilton, MT). MAb A57B9 against the chlamydial heat shock protein-60 (hsp60), was generously provided by Dr. Richard Morrison (University of Arkansas for Medical Sciences, Little Rock, AK). Antibodies to chlamydial LPS and eukaryotic actin (clone C4) were obtained from Chemicon International (Billerica, MA). TOPRO-3 (monomeric cyanine nucleic acid stain), and secondary antibodies conjugated to Alexa Fluor 488 and Alexa Fluor 568 were obtained from Invitrogen (Eugene, OR). Myriocin, fumonisin B1, dihydroceramide, sphingosine, 3-β-(2-diethylaminoethoxy)-androstenone HCl (U18666A), and brefeldin A were obtained from BioMol International (Plymouth Meeting, PA). Recombinant human IFN-γ was purchased from BD Biosciences (San Jose, CA). Golgicide A was kindly provided by Dr. David Haslam (Washington University School of Medicine, St. Louis, MO).

### Cell culture and propagation of chlamydiae

*C. trachomatis* serovar E (provided by Dr. Harlan Caldwell) and *C. trachomatis* serovar B (provided by Dr. Ted Hackstadt) were propagated in HEp-2 cells (ATCC, Manassas, VA) and elementary bodies (EBs) were purified by Renografin gradient centrifugation as previously described [36]. HEp-2 cells were maintained in Iscove's DME medium supplemented with 12.5 mM HEPES, 10% (vol/vol) FBS, and 10 µg/ml gentamicin, and grown at 37°C with 5.5% CO_2_. CHO-K1, LY-B, and LY-B/LCB1 cells, obtained from Dr. Kentaro Hanada (National Institute of Infectious Disease, Tokyo, Japan), were maintained in Ham's F12 medium supplemented with 10% (vol/vol) FBS, and 10 µg/ml gentamicin at 37°C with 5.5% CO_2_. Cells were infected by incubating monolayers with *Chlamydia* EBs at a multiplicity of infection (MOI) of 0.2 or 5 for 1 hr at 37°C, washed and incubated in fresh culture medium for the times indicated.

### Confocal microscopy

For immunofluorescence analyses, infected cells were fixed and permeabilized for 1 min with cold methanol. Cells were then incubated with the indicated primary and fluorophore-conjugated secondary antibodies, labeled with the nucleic acid stain TOPRO-3, and mounted in ProLong Anti-Fade (Invitrogen), as previously described [14]. Images were acquired using a Zeiss LSM510 Meta laser scanning confocal microscope (Carl Zeiss Inc., Thornwood, NY) equipped with a 63X, 1.4 numerical aperature Zeiss Plan Apochromat oil objective. Confocal Z slices of 0.5 µm were obtained using the Zeiss LSM510 software.

### Analysis of inhibitors

One hour post infection (pi), infected HEp-2 cells were incubated with medium containing inhibitors and the effects on inclusion development were determined by immunofluorescence, Western blot analysis, and infectivity assays, when indicated. To quantify the disruption of inclusions, one hundred infected cells were scored by fluorescence microscopy as indicated. Data are presented as the mean percent of disrupted inclusions. To quantify the number of inclusions per cell, one hundred infected cells were scored by fluorescence microscopy at 16 hr pi and presented as the mean number of inclusions per infected cell.

### Infectivity assays

Infected monolayers cultured in the presence of myriocin or IFN-γ were scraped from culture dishes, and supernatant and cells were analyzed to determine the number of infectious forming units (IFU) per ml (per 7.5×10^5^ infected cells). Data are presented as the mean+/−standard error of mean (s.e.m.) from one of three representative experiments.

### SDS-PAGE and immunoblotting

At the times indicated, infected monolayers were dissolved in Laemmli buffer and equivalent protein concentrations were analyzed by 10% SDS-PAGE. Western blots were probed with antibody to chlamydial OmcB, and antibody to host cell actin, which served as a loading control.

### Induction of persistence

HEp-2 cells were pretreated with 1 ng/ml IFN-γ for 48 hr prior to infecting with *C. trachomatis* B. Infected cells were then cultured in the presence of 1 ng/ml IFN for 48 hr, IFN was subsequently removed, and cells were incubated for an additional 48 hr with fresh culture medium with or without 25 µg/ml myriocin or 10 µM U18666A. At the indicated time points, inclusion development and infectivity were analyzed by immunofluorescence analysis and infectivity assays, respectively.

### Transmission electron microscopy

For ultrastructural analysis, infected HEp-2 cells were fixed in 2% paraformaldehyde/2.5% glutaraldehyde (Polysciences Inc., Warrington, PA) in 100 mM phosphate buffer, and processed as described previously [14].
